# Influence of Derecho and Management Disturbances on Ground-Dwelling Arthropods

**DOI:** 10.3390/biology15130984

**Published:** 2026-06-23

**Authors:** Jillian E. Wilson, Jordan M. Marshall

**Affiliations:** Department of Biological Sciences, Purdue University Fort Wayne, Fort Wayne, IN 46809, USA

**Keywords:** windstorm, salvage logging, diversity

## Abstract

Disturbance events and subsequent management practices significantly shape the ecological legacies of affected sites. This study evaluated the impacts of a 2022 derecho and the subsequent forest management on forest structure and arthropod diversity by comparing affected forests at Fogwell Forest Nature Preserve and Fox Island County Park with control forests at Blue Cast Springs and Hammer Wald Nature Preserves. Arthropod communities were sampled using pitfall traps, while forest structure was assessed through detailed surveys of understory, midstory, and overstory vegetation. Results indicated a decrease in overall arthropod diversity across all sites since 2016, variably attributed to forest maturation, climatic variability, and the 2022 disturbance, with some taxa showing declines, such as Formicidae and Curculionidae. Fogwell exhibited a significant decline in arthropod diversity, likely linked to the derecho, while Fox Island’s diversity aligned more closely with undisturbed control sites. Notable midstory reductions were observed across sites over time, especially at Fox Island, due to harvest and storm impacts. Meanwhile, overstory diversity varied between properties. Regression modeling revealed that forest management practices at Fox Island may have mitigated the disturbance’s effects, aiding arthropod recovery. All in all, these findings highlight the importance of forest management strategies in influencing biodiversity and ecological recovery post-disturbance.

## Simple Summary

Windstorm damage and forest harvesting directly influence forest composition and structure. This study evaluated the changes to arthropod abundance and diversity following a derecho (a rapidly moving, straight-line windstorm with thunderstorms) and subsequent forest harvesting. These changes were compared to pre-disturbance studies and sites not impacted by the storm. Overall, there was a decrease in arthropod diversity due to combinations of time, forest maturation, and the disturbances (storm and harvesting). The site with both storm and harvesting aligned with the expected arthropod diversity found at the control sites. The harvesting post-storm may have mitigated some of the storm effects, aiding in arthropod community resiliency.

## 1. Introduction

Forest ecosystems are dynamic systems shaped by complex environmental processes, including disturbance. Disturbance is defined as a discrete event in time and space that disrupts the structure of ecosystems, communities, or populations, thereby altering resource availability or environmental conditions [1]. The characteristics of disturbance regimes (i.e., scale, intensity, frequency) collectively determine ecological outcomes [2]. Both abiotic disturbances, including droughts, wildfires, and windstorms, and biotic disturbances, such as invasive species, pathogens, and insect outbreaks, have increased in frequency and severity due to ongoing climate change [3,4,5,6]. The ecological consequences of these disturbances are often mediated by legacy effects, which are persistent physical and biological structures that remain after an event [7]. Recognizing these legacy effects is essential for evaluating resilience and recovery in disturbed forests, as they can exert lasting influences on forest recovery trajectories and arthropod community composition [8]. The use of indicator species is a widely adopted method for evaluating ecosystem responses. Ground-dwelling arthropods have emerged as effective indicators in forested landscapes due to their sensitivity to subtle changes in habitat structure and their suitability for efficient, large-scale sampling [9,10,11,12].

In addition to natural disturbance regimes, anthropogenic activities such as forest management introduce additional sources of disturbance that can significantly alter post-disturbance ecosystems. Salvage logging, commonly implemented after windstorms or wildfires to recover economic value and reduce perceived hazards [13], modifies habitat structure by reducing coarse woody debris (CWD), altering soil properties, and changing microclimatic variables such as light, temperature, and moisture [14]. These habitat changes frequently result in declines in ground-dwelling taxa due to reduced resource availability and increased soil disturbance [14]. Conversely, some generalist taxa may benefit from increased habitat openness and greater vegetation heterogeneity [15]. The ecological impacts of salvage logging are therefore highly context-dependent, varying according to disturbance severity, logging intensity, and the time elapsed since disturbance [14].

Accurate assessment of ground-dwelling arthropod communities in disturbed and managed forests depends on the selection of appropriate sampling methodologies. Pitfall trapping is the most widely used technique because of its efficiency, cost-effectiveness, and minimal soil disturbance [16]. However, both capture rates and observed community composition are strongly influenced by sampling design parameters. For example, trap diameter affects capture efficiency, with intermediate-sized openings generally yielding higher catches [17]. Installing traps flush with the soil surface further enhances performance in forested environments [18]. The choice of preservative is also significant: ethylene glycol solutions are effective [19] but toxic, which limits their suitability for ecological studies [20]. Propylene glycol is a less toxic alternative and may influence capture rates through behavioral mechanisms [21,22]. Minimizing human activity near traps is essential, as repeated disturbance can affect capture outcomes [23].

Interpreting pitfall trap data requires careful consideration of both habitat structure and methodological limitations to ensure robust ecological inference. Litter depth and vegetation complexity can reduce trapping efficiency by blocking entrances and increasing the likelihood of arthropod escape [24]. Trap spacing, diameter, and the type of collecting fluid also influence observed measures of abundance, richness, and community composition [25,26]. Because pitfall traps provide a composite measure of arthropod abundance and activity, data interpretation should be approached with caution, particularly in heterogeneous habitats [27]. Structural elements of the forest, especially CWD, have a substantial impact on arthropod assemblages. Reductions in CWD are associated with declines in habitat complexity and shifts in community composition [28]. Integrating these factors is essential for generating reliable insights into the effects of forest disturbance and management on arthropod communities.

Considering these methodological and ecological factors, this study examines the effects of natural disturbance and subsequent management on ground-dwelling arthropod communities by sampling four forested sites: two impacted by a 2022 derecho (i.e., a rapidly moving, straight-line windstorm typically with heavy showers or thunderstorms) and the other two serving as controls. Arthropod community composition and forest structural characteristics were compared among disturbed and reference sites, as well as with pre-disturbance datasets, to assess legacy effects associated with canopy loss and structural change. The study specifically investigated how disturbance severity and post-disturbance management influenced arthropod biodiversity and community composition, testing the hypothesis that disturbance triggers taxon-specific responses and reduces overall arthropod biodiversity. While certain families are likely to be positively influenced by the disturbances, we expect overall diversity to decline due to alterations in forest structure and composition.

## 2. Materials and Methods

**Figure 1:**
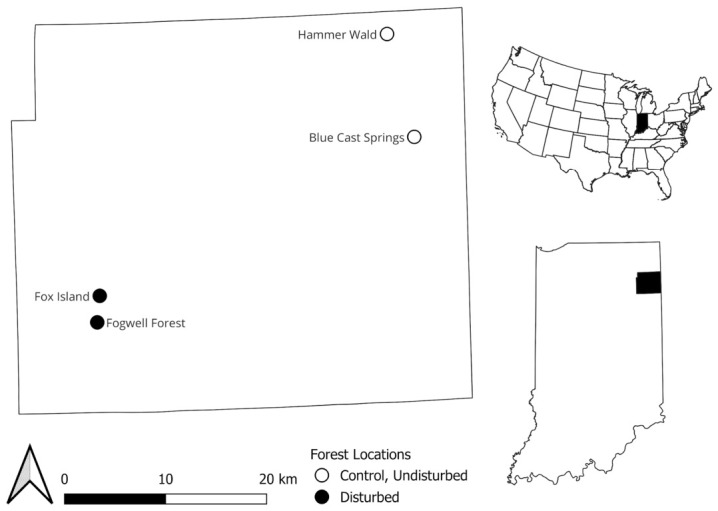
Locations of study sites. Disturbed properties were impacted by a derecho in 2022. Fox Island was impacted by selective harvesting following the derecho. Insets: United States with Indiana highlighted, Indiana with Allen County highlighted.

This study was conducted at four properties: three managed by ACRES Land Trust (Blue Cast Springs Nature Preserve, Fogwell Forest Nature Preserve, and Hammer Wald Nature Preserve) and one by Allen County Parks (Fox Island County Park), Allen County, Indiana, USA (Figure 1). These properties were selected based on prior arthropod and plant data collected in 2016 [29] and their recent exposure to a derecho disturbance event. Three of the properties were designated as state nature preserves (Blue Cast Springs, Fogwell Forest, and Fox Island), while one was privately protected but not state-designated (Hammer Wald). Fogwell Forest and Fox Island were impacted by a derecho on 13 June 2022, which experienced wind gusts at 157.7 kph. Blue Cast Springs and Hammer Wald were not impacted by this storm. Soil types at the four sites were mostly silt loam to silty clay loam, but with different definitions at each site (Blue Cast Springs: Nappanee silty clay loam and St. Clair silty clay loam; Fogwell Forest: Blount silt loam, ground moraine; Fox Island: Lenawee silty clay loam and Chelsea fine sand; Hammer Wald: Glynwood silt loam). These sites represent a mix of management types and disturbance histories, allowing for the examination of different forest conditions. Most notable with this project, Fox Island experienced a selective harvest to salvage merchantable logs in response to derecho damage, while minimal trail maintenance was performed at Fogwell Forest. Within each property, ten survey plots were established, with four randomly selected and six at locations that had been previously surveyed for arthropods or forest composition. The randomly selected plots were spaced 25.26 m apart on a grid within the forest boundaries (matching Myers and Marshall [29]), and their locations were mapped using QGIS (version 3.38.3). Fox Island plots were placed on a larger spaced grid of 31.62 m apart, similar to previous forest composition surveys. Due to accessibility limitations, only six plots were surveyed at Fox Island, with three randomly selected and three selected randomly from prior woody plant surveys.

### 2.1. Arthropod Surveys

Arthropod communities were surveyed using pitfall traps to capture ground-dwelling arthropods. At each plot center, three pitfall traps were established 1 m apart. These traps were ideally arranged in a straight line along the same contour, avoiding features that could influence arthropod movement, such as woody debris. If obstacles like woody debris or standing trees interfered with straight-line placement, traps were relocated, but with 1 m spacing to the nearest trap maintained. Each pitfall trap consisted of a plastic drinking cup (8 cm × 13 cm), buried such that the lip of the cup was flush with the soil surface (Figure S1). A 15 cm × 15 cm square of chicken wire was placed over the trap opening to prevent disturbance by wildlife, and a 20 cm Styrofoam plate was suspended 3 cm above the trap on 16d common nails to protect against rain and other environmental influences during trapping cycles.

To minimize potential bias from soil disturbance, traps were closed for seven days following initial installation, as pitfall trapping is a passive technique. During this period, the Styrofoam plate was pressed flush with the trap, and nails were used to secure it in place. After reopening the traps, 75 mL of 50% propylene glycol was added to each cup to act as both a killing and preserving agent. Pitfall traps were left open for seven days, and upon retrieval, all materials were removed. The pitfall traps from each plot were pooled in a container, and the number of intact traps (i.e., wildlife did remove some traps) was recorded to calculate the trapping effort (arthropods per trap).

In 2024, arthropod data were collected during two trapping cycles, with the first being set from 15 to 29 May 2024 and the second from 2 to 16 August 2024. During each cycle, traps were left open for seven consecutive days to ensure adequate sampling effort. At Fogwell Forest, pitfall trap data collection was restricted to eight plots per cycle due to environmental constraints (i.e., flooding) and trap malfunctions (i.e., setup errors). Specific plots sampled at Fogwell Forest varied by cycle, with plots 4 and 10 being excluded during the first cycle, and plots 7 and 9 being excluded during the second. Arthropods were identified to the family level when possible, or to the order level for soft-bodied arthropods that deteriorated in the traps. While many authors present a narrower definition of ground-dwelling arthropods [30,31], excluding families as “by-catch,” we included all arthropod taxa with ground-level activity necessary for capture in passive pitfall traps, regardless of occurrence frequencies or habitat preferences. This followed the data collection by Myers and Marshall [29].

### 2.2. Plant Surveys

Plants were categorized into three defined strata: understory, midstory, and overstory, which characterized the forest structure and organization. Understory plant percent ground cover (≤2 m in height) was estimated within a 1 m^2^ quadrat centered on each pitfall trap. Plant species were grouped into functional categories: herbaceous forbs, graminoids (grasses and sedges), trees, vines, shrubs, and ferns. The relative abundance of each functional group was recorded as the percentage of total cover in each quadrat, and a mean was calculated for the plot. Shannon’s diversity index was calculated for each plot based on the proportion of each group as:

(1) $$H^{\prime }=\;-\sum_{i=1}^{S}p_{i}lnp_{i}$$

where *p_i_* is the proportion of the *i*th functional group. Richness (S) was the count of different functional groups encountered.

For midstory plants (>2 m in height, ≤8 cm diameter at breast height [DBH, 1.3 m above the soil surface]), species identification and stem counts were conducted within a 25 m^2^ circular plot. Overstory plants (>8 cm DBH) were sampled within a 125 m^2^ circular plot at the plot center, where individual plants were identified to species and counted. Basal area (m^2^/ha) in each plot by species was estimated using a Jim-Gem Cruz-All with a 10-factor basal area (Forestry Suppliers, Jackson, MS, USA). Understory plant surveys were conducted at the end of the first arthropod trapping cycle, and midstory and overstory survey data collection began on 24 June 2024 and ended in early July 2024.

To quantify the relative importance of each overstory plant species, relative importance values (RIV) were calculated using the following formula:

RIV = (relative frequency + relative density + relative dominance)/3(2)

where relative frequency was calculated as the frequency of a species divided by the sum of all frequencies, where frequency was the percentage of 125 m^2^ circular plots where a species occurred × 100; relative density was the count of stems of a species within the 125 m^2^ circular plots divided by the sum of stems across all species × 100; and relative dominance as the basal area of a species divided by the total sum of basal area × 100.

### 2.3. Environmental Data

Environmental variables were assessed at each plot to characterize habitat conditions. Percent canopy cover was measured at plot centers using a concave spherical densiometer (Forestry Suppliers, Jackson, MS, USA) in four cardinal directions, and the mean was calculated for the plot. Percent slope was measured with a clinometer (Suunto, Vantaa, Finland) at the soil surface adjacent to each trap, and a plot mean was calculated. Fine woody debris (FWD, woody matter ≤ 1 cm in diameter) was counted within a 4 m^2^ quadrat centered on the plot. Litter depth was measured to the nearest 0.5 cm in each quadrant of the FWD quadrat using a meter stick. Additionally, in the 125 m^2^ circular plot used for overstory surveys, coarse woody debris (CWD, woody matter ≥ 10 cm in diameter at one end) was measured. CWD length was recorded at each entry and exit point of the plot, and volume was calculated using the formula:

CWD = ([*l* + *s*]/2) *L* (3)

where *l* is the area of the large end (m^2^), *s* is the area of the small end (m^2^), and *L* is the length of the log (m). All environmental data were collected between 25 June 2024 and early July.

### 2.4. Data Analysis

To calculate arthropod diversity, the Shannon diversity index was calculated for each plot and trapping cycle using Equation (1). Richness was the count of arthropod families captured. Arthropod and plant community diversity, as well as environmental variables, were compared between forests and between years using analysis of variance (ANOVA) with Tukey’s HSD post hoc test. ANOVA was also used to compare arthropod richness, diversity, and abundance between properties, with month included as a block to account for the trapping cycle. Nonmetric multidimensional scaling (NMDS) was used to visually compare Bray–Curtis dissimilarity between properties, with environmental factors plotted as joint vectors if their R^2^ was >0.1. Permutational multivariate analysis of variance (PERMANOVA) was used to identify differences in properties based on Bray–Curtis dissimilarity used in the NMDS with the *adonis2* function in the package ‘vegan’ (version 2.7-5) with post hoc pairwise comparisons with the *pairwise.adonis2* function in package ‘pairwiseAdonis’ (version 0.4.1) followed by a Holm *p*-value adjustment.

Bray–Curtis dissimilarity between plots within a forest was calculated for each property to identify heterogeneity in both composition and structure. Compositional heterogeneity was calculated as dissimilarity between plots in pooled understory, midstory, and overstory data. Structural heterogeneity was calculated as dissimilarity between plots in pooled environmental variables.

To understand the relationships between disturbance and time with the arthropod communities, a multiple regression model was developed using the 2016 arthropod diversity and environmental data via reverse variable selection. This model was applied to the 2024 environmental data to provide expected arthropod diversities for each property. The observed and predicted 2024 arthropod diversities were plotted to visualize the changes due to time and disturbance. The time between 2016 and 2024 data collection includes other natural disturbances and successional responses by the forest. None of the sites received any active anthropogenic management and did not appear to experience any other stand-level disturbances, other than the 2022 derecho. As forests are not stagnant, it is probable that single-tree-level disturbances or mortality occurred within all the sites, creating localized gaps as a standard part of stand dynamics [32]. As Blue Cast Springs and Hammer Wald were not impacted by the derecho, these sites represented controls, and differences in observed and predicted arthropod diversity should be influenced by time (2016 to 2024). Fogwell was impacted by the derecho, with post-storm management limited to clearing trails of debris, and differences in arthropod diversity should be influenced by time and storm. Fox Island was also impacted by the derecho with much more intensive post-storm management that included relatively large-scale selective harvesting of trees throughout many areas of the park, and differences in arthropod observed and predicted diversity should be influenced by time, storm, and harvesting. Dependent variables were tested with the Shapiro–Wilk test to ensure no violation of the assumption that data were drawn from a normally distributed population. All analyses were conducted in R (version 4.4.1).

## 3. Results

### 3.1. Plant Surveys

**Figure 2:**
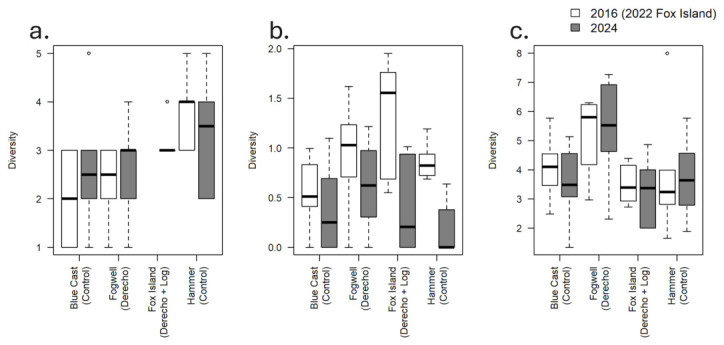
Understory plant functional group (**a**), midstory species (**b**), and overstory species (**c**) diversity between years. Note, there was no previous understory data collected at Fox Island, while midstory and overstory data were collected in 2022 prior to the derecho.

**Figure 3:**
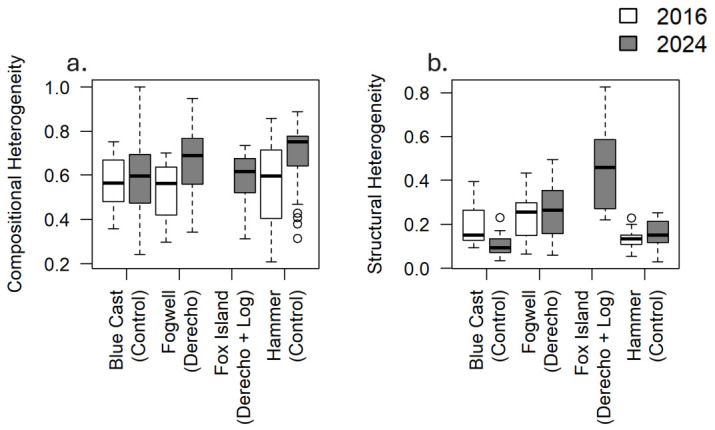
Heterogeneity as Bray–Curtis dissimilarity within properties for (**a**) composition (pooled understory, midstory, and overstory data) and (**b**) structure (pooled environmental data). Note, there was no previous understory or environmental data collected at Fox Island.

We encountered a total of 23 overstory species and 18 midstory species amongst the four properties surveyed in 2024 (Tables S1 and S2). Based on RIV, we classified all forested properties, except for Fox Island, as a Sugar Maple forest type [33] (Table S3), which was consistent with classifications from 2016 [29]. Fox Island did not align with a forest type defined by Eyre [33] as overstory species presence varied heavily between plots, with *Celtis occidentalis* L. being more abundant in wetter zones and other species such as *Ulmus americana* L., *Prunus serotina* Ehrh., and *Carya glabra* (Mill.) Sweet is found in the upland. Standing dead in the overstory ranked amongst the top five across properties and between years. Understory diversity varied significantly between properties (F_3,47_ = 5.50, *p* = 0.003; Figure 2a) but not years (F_1,47_ = 0.37, *p* = 0.546), with Blue Cast and Fogwell Forest increasing and Hammer Wald decreasing from previous surveys. However, due to the low resolution of the Shannon index for the diversity of the observed functional groups, understory diversity may not provide a meaningful metric. Midstory vegetation exhibited significant declines between surveys in both diversity and richness over time (F_2,52_ = 34.149, *p* < 0.001; F_2,52_ = 34.15, *p* < 0.001, respectively; Figure 2b), a trend consistent across all properties, likely reflecting either a loss of species or reduced recruitment. In contrast, both overstory diversity and richness varied significantly between properties (F_7,52_ = 2.87, *p* = 0.013; Figure 2c) but not between years (F_2,52_ = 0.762, *p* = 0.471). Plant community composition heterogeneity resulted in significant differences between years (F_1,188_ = 15.97, *p* < 0.001; Figure 3a) and between properties (F_3,188_ = 3.19, *p* = 0.025), underscoring the influence of local site conditions on forest composition and structure.

### 3.2. Environmental Data

**Figure 4:**
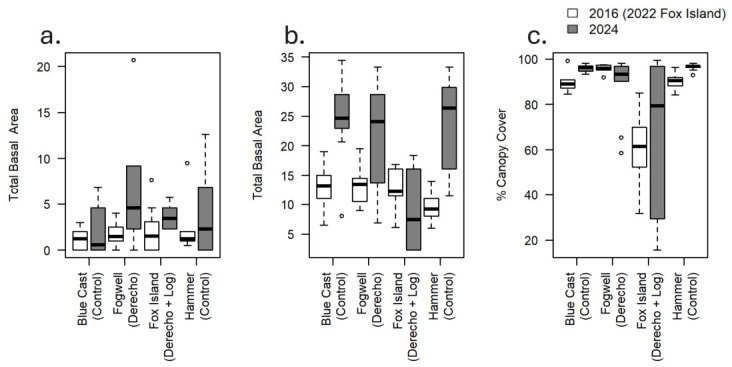
Standing dead basal area (m^2^/ha) (**a**), standing live basal area (m^2^/ha) (**b**), and percent canopy cover (**c**) between sites and years.

**Figure 5:**
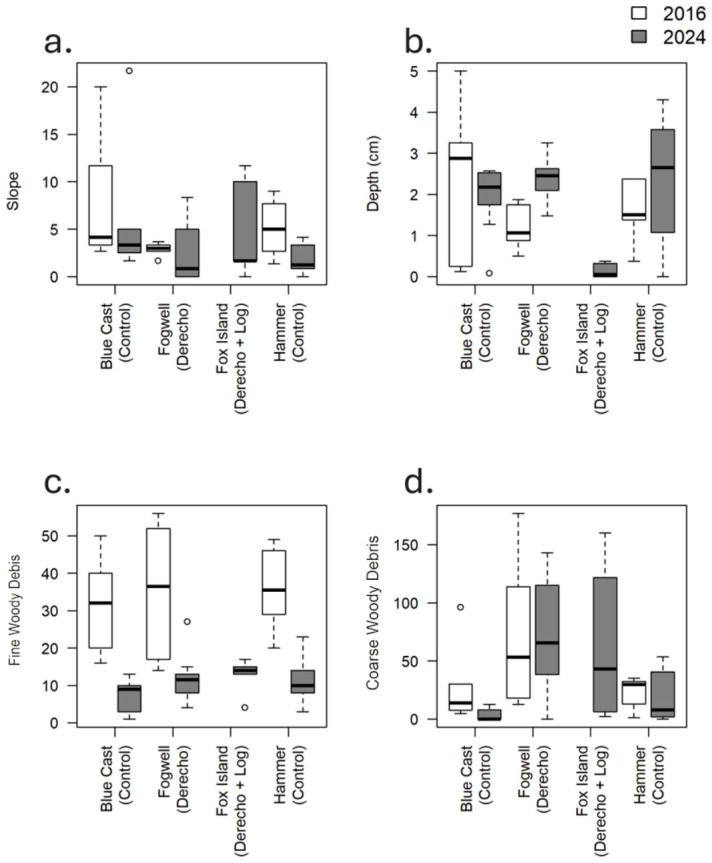
Percent slope (**a**), leaf litter depth (**b**), fine woody debris (count/m^2^) (**c**), and coarse woody debris (m^3^/ha) (**d**) between sites and time.

Environmental variables such as canopy cover, CWD, and litter depth differed significantly between the properties in 2024 (F_3,61_ = 10.50, *p* < 0.001; F_3,61_ = 7.29, *p* < 0.001; F_3,61_ = 6.13, *p* < 0.001; respectively). Canopy cover was greatest at Hammer Wald (Figure 4c), coarse woody debris was greatest at Fox Island and Fogwell Forest (Figure 5d), and litter depth was consistently lower at Fox Island compared to other sites (Figure 5a). Additionally, FWD, while low across all sites, differed significantly among properties (F_3,61_ = 76.73, *p* < 0.001; Figure 5b), highlighting a significant temporal shift in forest structure.

Changes in environmental conditions between 2016 and 2024 further underscore the dynamic nature of these sites. Canopy cover trends over time showed a decrease at Fogwell Forest, an increase at Hammer Wald, and stability at Blue Cast Springs and Fox Island (Figure 4c). FWD continued to decline significantly across all properties from 2016 to 2024 (F_1,61_ = 76.73, *p* < 0.001; Figure 5c). Standing dead basal area did not differ significantly overall, but it notably increased at Fox Island (Figure 4a). In contrast, the basal area of living trees remained relatively stable across all sites, except at Hammer Wald, where a significant increase was observed (Figure 4b).

Structural heterogeneity comparison resulted in significant differences among properties (F_2,188_ = 5.89, *p* = 0.003, Figure 3b), with Fox Island and Fogwell Forest being the most dissimilar between plots in terms of structure. Heterogeneity of the site also significantly differed between years (F_3,188_ = 66.41, *p* < 0.001), with Blue Cast Springs becoming more similar in 2024 than in 2016 and other properties remaining relatively similar over time.

### 3.3. Arthropod Surveys

**Figure 6:**
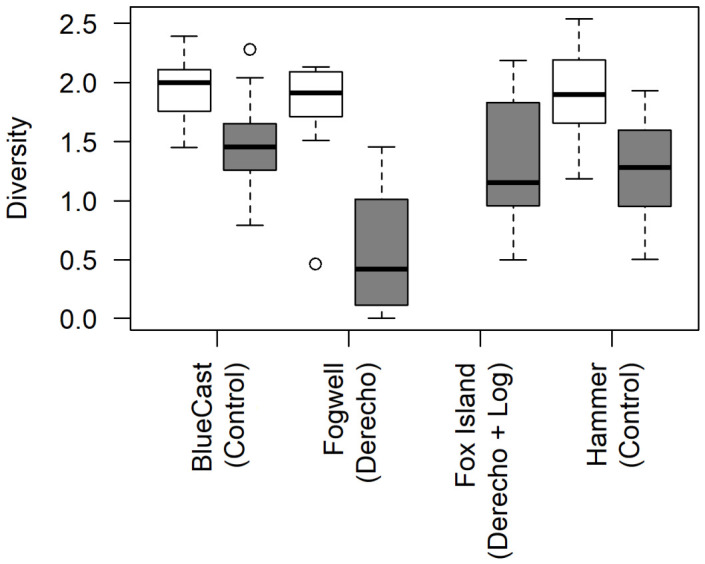
Arthropod diversity (Shannon index, H’) between sites and time (white boxes = 2016, gray boxes = 2024). Note, there was no capture data for Fox Island in 20216.

We encountered a total of 2239 insects from 51 families and 3359 non-insect arthropods from 5 orders and 5 classes (Table S4) across the four properties surveyed in 2024. Isopoda remained the most abundant taxon across sites and over time, with significant increases in Opiliones and Orthoptera families in 2024 (Table S4). Arthropod richness, diversity, and abundance in pitfall traps varied significantly between properties in 2024 (richness: F_3,87_ = 2.85, *p* = 0.042; diversity: F_3,87_ = 14.23, *p* < 0.001; abundance: F_3,87_ = 9.04, *p* < 0.001; Figure 6). Richness, diversity, and abundance followed similar patterns.

**Figure 7:**
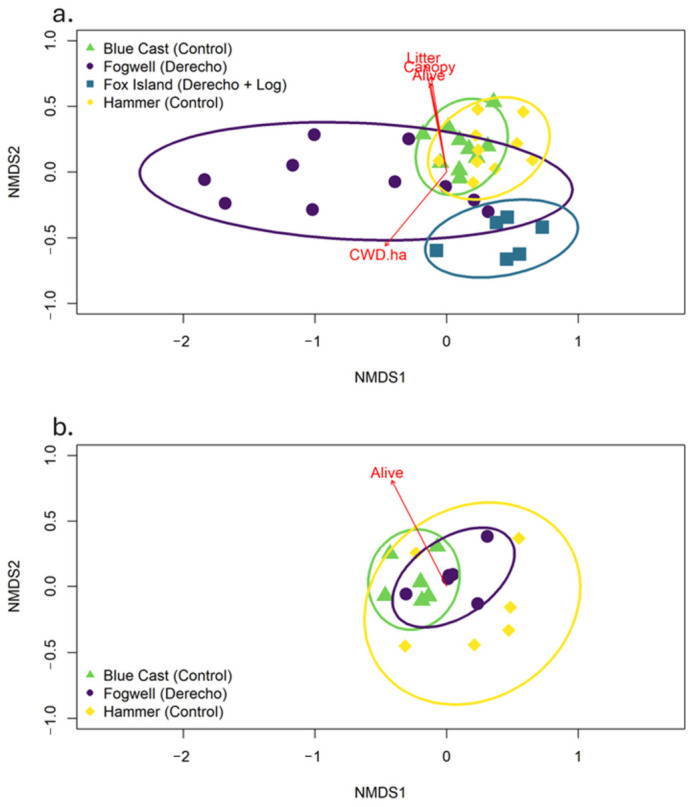
Nonmetric multi-dimensional scaling (NMDS) ordination for arthropod communities in 2024 (**a**) and 2016 (**b**). Red vector direction and length indicate the influence of environmental variables (R^2^ > 0.1) on dissimilarities. Ellipses represent 95% confidence intervals around plots. Note, there was no capture data for Fox Island in 2016.

Arthropod community NMDS ordinations were used to visualize dissimilarity between properties in 2024 and 2016. Each point represented an arthropod community at a plot within a property. Ordinations were assessed using stress values (a measure of how well the data fit the ordination output), distance (visualized similarity between sites based on how close or far apart they are), and vectors (direction and length indicate how strongly and directly environmental variables influence what was observed at each property). The NMDS ordinations revealed distinct community compositions, with low stress values in both years (2016: Stress = 0.11; 2024: Stress = 0.12; Figure 7). In 2016, the basal area of live trees was the primary environmental driver of arthropod communities, whereas in 2024, litter depth, CWD, and canopy cover became important predictors. There was a significant difference in the distance matrices for both 2024 and 2016 communities (F_3,32_ = 6.52, *p* = 0.001; F_2,15_ = 3.66, *p* = 0.004, respectively). In 2024, all four properties had significantly different distances using post hoc pairwise comparisons (Table S5). However, in 2016, Fogwell did not differ from Blue Cast or Hammer, while Blue Cast and Hammer significantly differed (Table S5).

**Figure 8:**
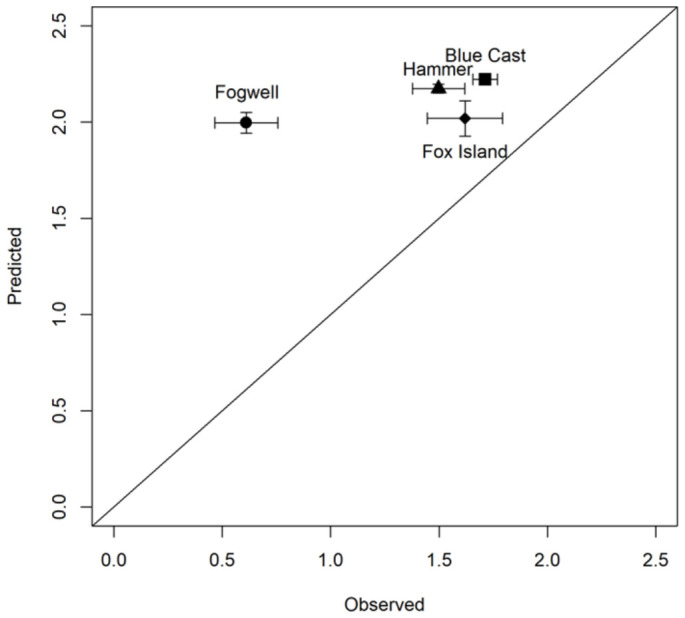
Observed versus predicted diversity (Shannon index, H’) of ground-dwelling arthropods. Mean site values are represented by unique markers with standard errors. The diagonal represents an isoline where observed equals predicted values.

To evaluate the impact of management practices on arthropod diversity at Fox Island, a regression model was created using 2016 arthropod diversity and environmental variable data available from Fogwell Forest, Blue Cast Springs, and Hammer Wald, which predicted 2024 arthropod diversity based on environmental variables collected in 2024 (Figure 8). The regression analysis started with the global model including CWD, FWD, alive tree basal area, dead tree basal area, percent canopy cover, and percent slope. Using reverse variable selection (i.e., backward elimination [34]), we removed independent variables that were not significantly adding to the explanation of the dependent variable variation. The final model only included CWD as an independent variable.

Arthropod Diversity = 2.235 − 0.003 × *CWD* (4)

where *CWD* was observed, coarse woody debris volume (m^3^/ha) within the plot (F_1,16_ = 6.84, *p* = 0.019, R^2^ = 0.30). Observed and predicted values were plotted for 2024 to visualize diversity trends at each property, including Fox Island, which lacked 2016 data for comparison. Values plotted on the isoline indicate equal observed and predicted values, while those above the isoline indicate over-prediction (greater expected diversity than observed). Control sites (Blue Cast Springs and Hammer Wald) were slightly over-predicted, while the model greatly over-predicted Fogwell Forest diversity, highlighting the impact of the derecho on arthropod populations. Fox Island aligned more closely with the control sites. This result may indicate that Fox Island’s current arthropod diversity does not appear different from other local forests based on CWD.

## 4. Discussion

Disturbance events, such as the derecho and salvage harvest observed in these study sites, have been shown to cause significant changes in forest structure and composition, which in turn can significantly influence arthropod communities [29,32]. Disturbances create a dynamic environment that benefits certain disturbance-adapted taxa while potentially harming others [35]. Our results provide insights into how the derecho and subsequent management altered forest structure, which led to shifts in both arthropod and plant communities. The results of arthropod abundance and diversity were collected at the family level, which requires conservative interpretation due to the variation that can exist in trophic level, ecological niche, and life history characteristics within an arthropod family. While some taxa, like Opiliones (harvestmen) and Orthoptera (Gryllidae and Gryllacrididae), increased in abundance, overall arthropod diversity declined, mirroring broader patterns of biodiversity response to disturbance in the short term. Changes in midstory vegetation further suggested that habitat restructuring influenced multiple trophic levels. Differences between managed and unmanaged sites highlight the role of post-disturbance management in shaping recovery trajectories.

Increases in Opiliones and Orthoptera abundance in 2024, particularly in sites affected by the derecho, were consistent with other studies documenting the proliferation of disturbance-adapted species [35]. Opiliones, for example, are known to increase in abundance in environments with increased CWD and altered litter layers, which provide additional shelter and hunting opportunities [36,37]. Similarly, Orthoptera tend to also increase in abundance in disturbed environments, where increased sunlight and ground vegetation support greater food availability and microhabitat complexity [38]. The high quantity of CWD, especially at Fox Island and Fogwell, likely provided critical habitat for ground-dwelling arthropods, supporting the persistence of these taxa in the altered forest structure, which supports our original hypothesis. These results suggest that disturbances can create conditions that benefit certain arthropod groups, particularly those adapted to exploit new, more heterogeneous environments [13,29,39].

Other taxonomic groups exhibited little change in dominance or abundance following the disturbance events. Between sites and years, isopods were consistently the most abundant group, except at Blue Cast in 2016, where they ranked third. Isopods play a crucial role in decomposition processes by breaking down leaf litter and contributing to nutrient cycling in forest ecosystems [40]. Their high abundance, even after disturbance, suggests that they are persistent and resistant to changes in habitat structure and microclimatic conditions [41]. One possible explanation for their persistence is their reliance on moist microhabitats, which may have been maintained by the accumulation of CWD and deep litter layers in some disturbed areas. Additionally, isopods are known to thrive in environments with increased availability of organic matter, which can result from canopy openings and subsequent increases in primary production following disturbances [41]. The ability of isopods to exploit decomposing wood and leaf litter likely contributed to their continued dominance, even as other arthropod taxa declined in response to habitat changes.

Despite these positive and neutral responses from some taxa, other groups experienced major declines. For example, Curculionidae (weevils) ranked second or third in abundance across sites in 2016 but decreased to seventh or lower in 2024. Similarly, Formicidae (ants) were consistently in the top five most abundant taxa in 2016 but fell out of that ranking by 2024. Weevils, which primarily rely on plant material for food and larval development, may have been negatively impacted by the observed decline in midstory vegetation diversity across all sites [14,42]. Many weevil species are host-specific, with larvae developing inside plant tissues, including leaves, stems, and seeds. A reduction in plant diversity, particularly in the midstory, likely decreased the availability of host plants, which could explain the decline in weevil captures over time. Additionally, the overall changes in canopy cover may have increased exposure to desiccation and predation, further contributing to their reduced abundance [43]. Ants also showed a substantial decline, which may be linked to changes in microhabitat conditions, particularly the drier field season in 2024. Many forest ant species are highly dependent on soil moisture and organic matter for nest construction and foraging [39]. The reduced litter depth and lower canopy cover at Blue Cast and Fox Island could have led to drier soil and leaf litter conditions, making the environment less hospitable than for ground-dwelling ant species to construct their nests. Drier conditions can also impact weevil and ant activity levels, as many species reduce foraging during periods of high heat and low humidity to avoid desiccation. There was a notable difference in growing season rainfall between 2016 and 2024, resulting in measurable drought conditions [44]. While we did not include drought as a quantitative variable, it is important to include it in the interpretation. This may explain the lower capture rates in 2024, as ants may have altered their activity patterns or shifted to deeper soil layers, making them less likely to be sampled [45]. Furthermore, declines in other arthropod taxa due to disturbance and drought conditions may have resulted in reduced prey availability, further impacting their abundance [41]. These results suggest that while some taxa, like isopods, were able to persist or even thrive post-disturbance, others, particularly those sensitive to microclimatic changes, were negatively affected by the combined effects of disturbance and drier field season [46].

Analysis of the broader ground-dwelling community resulted in an overall pattern across properties of declining arthropod diversity, a trend that is consistent with broader patterns of insect decline documented in recent decades [47] but should be evaluated further. This decline was especially pronounced at Fogwell, where disturbance was followed by minimal management intervention. Multiple factors likely contributed to this trend, including habitat loss, climate change, and altered resource availability [48]. Studies have shown that arthropod populations worldwide are experiencing significant declines due to anthropogenic pressures, with habitat fragmentation, pesticide use, and climate fluctuations playing key roles [49]. The particularly dry field season in 2024 may have exacerbated these declines, as many arthropod taxa are highly sensitive to moisture availability and microclimate stability [39]. Previous studies indicate that drought conditions can reduce arthropod abundance by altering vegetation cover, limiting food availability, and increasing mortality due to desiccation [50,51]. The reduction in litter depth at several sites, which serves as a crucial moisture-retaining microhabitat characteristic for many ground-dwelling arthropods, may have further contributed [52]. The broader implications of these findings suggest that without active management and conservation efforts, forest arthropod communities may continue to decline [48].

The observed decline in midstory vegetation diversity across derecho-disturbed properties suggests that disturbance events, particularly those that cause significant canopy disruption, can hinder regeneration and reduce species richness, especially in the midstory and understory layers [43]. The suppression of midstory regeneration, especially in sites with minimal management, indicates that the lack of active management following disturbances can slow forest recovery, further supporting the notion that disturbances may have long-term negative effects on overall forest biodiversity [15]. Although less significant than in the derecho-affected areas, midstory declines were also noted at control properties, likely due to smaller disturbances (e.g., single tree mortality) since the initial survey in 2016 and the continued succession of those sites [32]. These declines were consistent with the idea that suppressed regeneration can lead to reduced arthropod abundance at those sites [43].

Forest management can play a key role in mitigating some of the detrimental impacts of large or high-intensity natural disturbance events. At Fox Island, where management was implemented in the form of a salvage harvest, the community composition and diversity of both plants and arthropods remained more like those of control sites. These results align with research indicating that forest management can buffer the negative effects of disturbance by maintaining habitat heterogeneity and promoting resilience [34,53]. For example, D’Amato et al. [53] found that post-disturbance salvage logging, when applied with ecological considerations, can enhance recovery by preserving key habitat structures. We hypothesize that Fox Island’s retention of structural complexity—particularly in the overstory and midstory layers—as well as its high structural heterogeneity, in general, may have helped stabilize arthropod communities by providing a diverse range of microhabitats and resources (i.e., CWD accumulation and declines).

Although environmental factors such as canopy cover, coarse woody debris (CWD), and litter depth did not show significant differences across sites, they contributed to fine-scale variation in structural and compositional heterogeneity. Our analysis of arthropod communities was conducted at the plot level, which could be argued to be pseudoreplication due to occurrences in the same forest site. However, structural and compositional heterogeneity in each forest was relatively high. Additionally, the NMDS ordination revealed that litter depth was positively correlated with arthropod abundance, particularly in the driest sampling time in August. The combined findings of site heterogeneity and changes in litter layers suggest that these factors create favorable microhabitats for certain arthropods, which may serve as refugia following disturbances [39]. Furthermore, increased leaf litter can enhance water retention and provide shelter for specific ground-dwelling taxa [54], while reductions in leaf litter may increase the vulnerability of prey species to predation [41].

## 5. Conclusions

In conclusion, the results supported our hypothesis that disturbance has both positive and negative effects on arthropod communities, depending on the taxa involved and the management strategies used. Disturbances such as derecho events create new habitat conditions that can benefit disturbance-adapted taxa, such as Opiliones and Orthoptera, and hinder stability-adapted taxa, such as Formicidae and Curculionidae, but generally lead to declines in overall arthropod diversity, particularly when management interventions are minimal. The evidence from Fox Island demonstrates that active management may have a potential mitigating influence on the negative impacts of disturbance by maintaining structural complexity and habitat heterogeneity [53]. However, the decline in midstory diversity and the reduction in arthropod richness across sites emphasize the need for long-term monitoring and adaptive management strategies to support forest recovery and enhance resilience. Additionally, such monitoring needs to be expanded to other taxonomic groups beyond arthropods to fully understand the interaction of natural disturbance and forest management. There are relationships between forest heterogeneity and arthropod community abundance and diversity, but there may be limitations to using arthropods as indicators, especially across different spatial scales [15,29,39]. Future research should continue to explore the functional roles of arthropod taxa and the complex interactions between disturbance, management, and diversity across different forest types. Expanding research across diverse forest ecosystems will be critical for developing conservation and management strategies that enhance forest resilience and biodiversity in the face of ongoing disturbances.

## Data Availability

Data are available in the Supplementary Materials.

## Supplementary Materials

The following supporting information can be downloaded at: https://www.mdpi.com/article/10.3390/biology15130984/s1, Table S1: Annotated species list from overstory surveys; Table S2: Annotated species list from midstory surveys; Table S3: Overstory species importance value ranking between sites and years; Table S4: Taxonomic list and count of arthropods collected. Table S5. Permutational multivariate analysis of variance (PERMANOVA) of property Bray-Curtis dissimilarity distance matrices post-hoc pairwise comparison results for 2016 and 2024 using pairwise.adonis2 function in package ‘pairwiseAdonis’ (version 0.4.1) with a Holm *p*-value adjustment. Figure S1. Diagram illustrating pitfall trap arrangement with 1 m spacing between individual traps (a) and installation (b).

## Abbreviations

The following abbreviations are used in this manuscript:

ANOVA	Analysis of variance
CWD	Coarse woody debris
DBH	Diameter at breast height
FWD	Fine woody debris
HSD	Honestly significant difference
NMDS	Non-metric multidimensional scaling
RIV	Relative importance value
