# Supplementary material for: Influence of Derecho and Management Disturbances on Ground-Dwelling Arthropods
Source: Biology (Basel). 2026 Jun 23;15(13):984. doi: 10.3390/biology15130984 (PMC13360023; doi:10.3390/biology15130984)
Supplement: Supplementary file 1 [file biology-15-00984-s001.zip › Wilson_Marshall_Figure_S1.pdf]

a.

b.

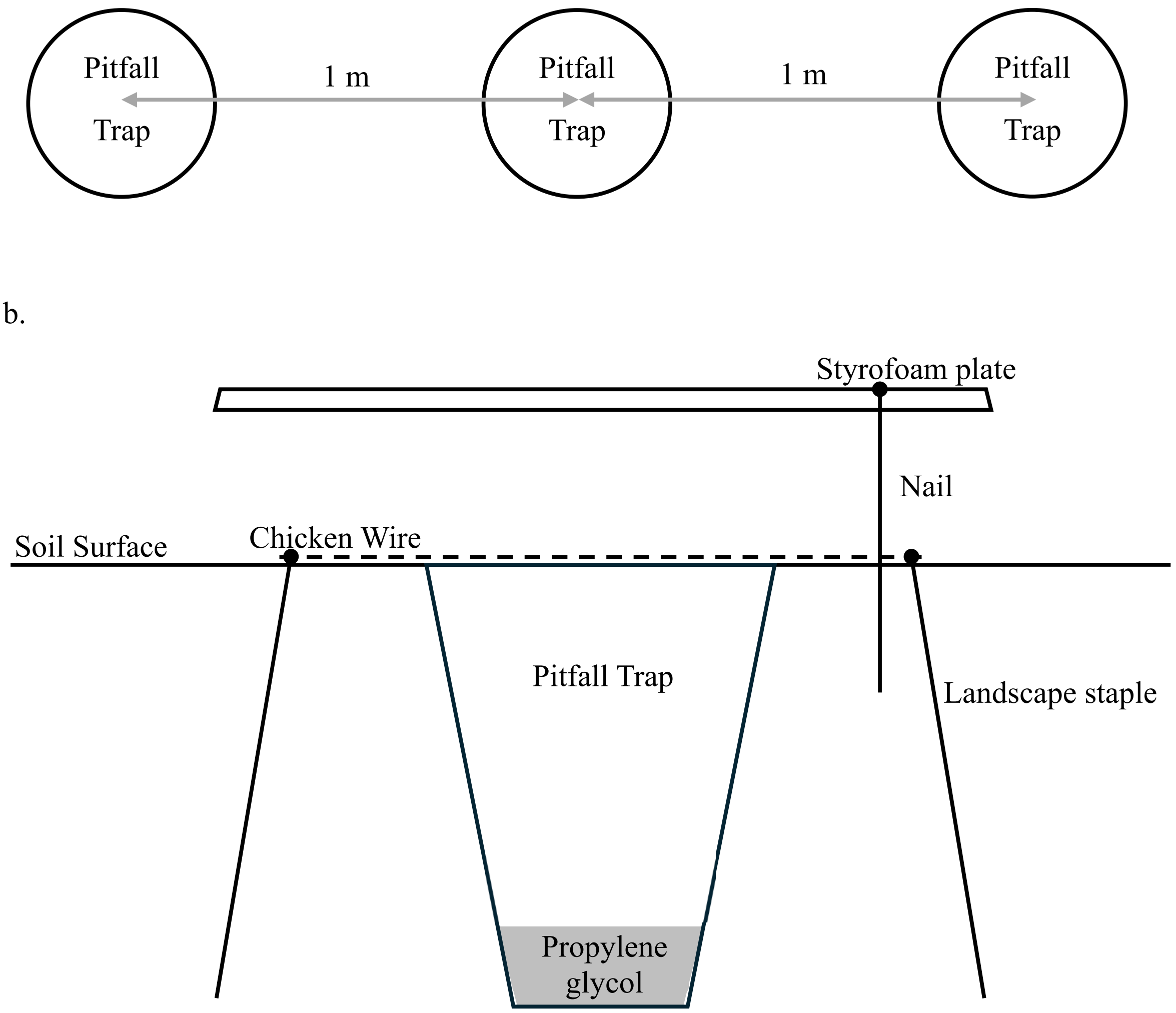

Figure S1. Diagram illustrating pitfall trap arrangement with 1 m spacing between individual traps (a) and installation (b).
