# Supplementary material for: Influence of Derecho and Management Disturbances on Ground-Dwelling Arthropods
Source: Biology (Basel). 2026 Jun 23;15(13):984. doi: 10.3390/biology15130984 (PMC13360023; doi:10.3390/biology15130984)
Supplement: Supplementary file 1 [file biology-15-00984-s001.zip › Wilson_Marshall_Table_S1.pdf]

Table S1. Annotated species list for overstory plants in Blue Cast Springs Nature Preserve (BLU), Fogwell Forest Nature Preserve (FOG), Fox Island County Park (FOX), and Hammer Wald Nature Preserve (HAM). Species binomial followed by three-letter property codes and year of collection.

ANNONACEAE
Asimina triloba L. – FOX (2022)

BETULACEAE
Ostrya virginiana (Mill.) K. Koch – BLU, HAM (2024); BLU, HAM (2016)

CANNABACEAE
Celtis occidentalis L. – FOX (2024); FOX (2022); FOG (2016)

FABACEAE
Robinia pseudoacacia L. – BLU, FOX (2024); BLU (2016)

FAGACEAE
Fagus grandifolia Ehrh. – BLU, FOG, FOX (2024); FOG (2016)
Quercus alba L. – BLU, FOG (2024); BLU, FOG (2016)
Quercus bicolor Willd. – BLU, FOG, HAM (2024); BLU, FOG, HAM (2016)
Quercus macrocarpa Michx. – FOX (2024)
Quercus rubra L. – BLU, FOG, FOX, HAM (2024); FOX (2022); BLU, FOG, HAM (2016)
Quercus velutina Lam. – BLU (2024); BLU (2016)

JUGLANDACEAE
Carya glabra (Mill.) Sweet – BLU, FOX, HAM (2024); BLU, HAM (2016)
Carya cordiformis (Wangenh.) K. Koch – FOX (2016)
Carya ovata (Mill.) K. Koch – BLU, FOG, HAM (2024); BLU, FOG, HAM (2016)
Juglans nigra L. – BLU, FOG, FOX (2024); FOX (2022) FOG, HAM (2016)

LAURACEAE
Sassafras albidum (Nutt.) News – FOX (2022)

MAGNOLIACEAE
Liriodendron tulipifera L. – BLU, FOG (2024); FOX (2022); FOG (2016)

MALVACEAE
Tilia americana L. – BLU, FOG, HAM (2024); BLU, FOG, HAM (2016)

OLEACEAE
Fraxinus pennsylvanica L. – FOX (2022)

ROSACEAE
Crataegus spp. L. – BLU (2024); HAM (2016)
Prunus serotina Ehrh. – FOX, HAM (2024); FOX (2022); HAM (2016)

SALICACEAE
Populus deltoides W. Bartram ex Marshall – FOX (2024); FOX (2022)

SAPINDACEAE
Acer negundo L. – FOX (2024); FOX (2022)
Acer nigrum L. – FOX (2022)
Acer rubrum L. – FOG (2024); BLU, FOG, HAM (2016)
Acer saccharinum L. – FOG, HAM (2016)
Acer saccharum Marsh. – BLU, FOG, HAM (2024); FOX (2022); BLU, FOG, HAM (2016)
Aesculus glabra Willd. – BLU, FOG (2024); FOX (2022); BLU, FOG (2016)

ULMACEAE
Ulmus americana L. – FOG, FOX, HAM (2024); FOX (2022); FOG, HAM (2016)
Ulmus rubra Muhl. – FOG, HAM (2024); FOG (2016)
