# Supplementary material for: Influence of Derecho and Management Disturbances on Ground-Dwelling Arthropods
Source: Biology (Basel). 2026 Jun 23;15(13):984. doi: 10.3390/biology15130984 (PMC13360023; doi:10.3390/biology15130984)
Supplement: Supplementary file 1 [file biology-15-00984-s001.zip › Wilson_Marshall_Table_S2.pdf]

Table S2. Annotated species list for midstory plants in Blue Cast Springs Nature Preserve (BLU), Fogwell Forest Nature Preserve (FOG), Fox Island County Park (FOX), and Hammer Wald Nature Preserve (HAM). Species binomial followed by three-letter property codes and year of collection.

ADOXACEAE
Sambucus nigra L. – FOX (2022)
Viburnum acerifolium L. – FOX (2022)
Viburnum prunifolium L. – FOG (2016); FOX (2022)

ANNONACEAE
Asimina triloba (L.) Dunal – FOG (2024)

BETULACEAE
Carpinus caroliniana Walter – FOG (2024), BLU, FOG (2016)
Corylus americana Walter – FOX (2022)
Ostrya virginiana (Mill.) K. Koch – BLU, HAM (2024); BLU, FOG, HAM (2016)

CANNABACEAE
Celtis occidentalis L. – FOX (2024); FOX (2022)

CAPRIFOLIACEAE
Lonicera maackii (Rupr.) Herder – BLU, FOX (2024); FOX (2022)
Lonicera tartarica L. – FOX (2022)

CORNACEAE
Cornus florida L. – FOX (2022)
Cornus drummonii L. – FOX (2024)
Cornus racemosa Lam. – FOX (2022)

ELAEAGNACEAE
Elaeagnus umbellata Thunb.. – FOX (2022)

FAGACEAE
Fagus grandifolia Ehrh. – FOG, HAM (2016)
Quercus rubra L. – HAM (2016)

HAMAMELIDACEAE
Hamamelis virginiana L. – FOX (2022)

JUGLANDACEAE
Carya cordiformis (Wangenh.) K. Koch – FOX (2022)
Carya glabra (Mill.) Sweet – BLU (2024), HAM (2016)
Juglans nigra L. – BLU, FOG, FOX (2024); FOX (2022) FOG, HAM (2016)

LAURACEAE

Lindera benzoin (L.) Blume – FOG, FOX (2024); FOX (2022); FOG (2016)
Sassafras albidum (Nutt.) Nees – FOX (2024); FOX (2022)

MAGNOLIACEAE
Liriodendron tulipifera L. – FOX (2022)

MALVACEAE
Tilia americana L. – FOG (2024), FOG (2016)

MORACEAE
Morus alba L. – FOX (2022)

OLEACEAE
Fraxinus pennsylvanica Marsh. – BLU, FOG, FOX, HAM (2024); FOX (2022); BLU, HAM (2016)
Fraxinus quadrangulata Michx. – FOG, HAM (2016)

ROSACEAE
Prunus serotina Ehrh. – FOX (2022); BLU (2016)
Rosa multiflora Thunb. – FOX (2024); FOX (2022)
Rubus spp. L. – FOX (2024)

SAPINDACEAE
Acer negundo L. – FOX (2022)
Acer saccharinum L. – BLU (2024); BLU (2016)
Acer saccharum Marsh. – FOG, HAM (2024); FOG, HAM (2016)
Aesculus glabra Willd. – FOG, FOX (2024); FOX (2022); FOG (2016)

ULMACEAE
Ulmus americana L. – FOG, FOX, HAM (2024); FOX (2022); FOG, HAM (2016)
Ulmus rubra Muhl. – FOG, HAM (2024); FOG (2016)
