# Supplementary material for: Influence of Derecho and Management Disturbances on Ground-Dwelling Arthropods
Source: Biology (Basel). 2026 Jun 23;15(13):984. doi: 10.3390/biology15130984 (PMC13360023; doi:10.3390/biology15130984)
Supplement: Supplementary file 1 [file biology-15-00984-s001.zip › Wilson_Marshall_Table_S3.pdf]

Table S3. Overstory species importance value ranking between sites and years. Top 5 species at each site and year shaded. Blue Cast Springs Nature Preserve (BLU), Fogwell Forest Nature Preserve (FOG), Fox Island County Park (FOX), and Hammer Wald Nature Preserve (HAM).

	BLU		FOG		FOX		HAM	
Species	2016	2024	2016	2024	2022	2024	2016	2024
Acer negundo					6	8		
Acer nigrum					16			
Acer rubrum	11		13	13			13	
Acer saccharinum			16				7	
Acer saccharum	2	2	1	2	3		2	1
Aesculus glabra	11	7	12	10	16			
Asimina triloba					16			
Carya cordiformis					9			
Carya glabra	9	6				3	9	8
Carya ovata	1	1	7	5	13		3	5
Celtis occidentalis			14		7	5		
Cornus florida					16			
Corylus americana					16			
Crataegus sp.		14					14	
Fagus grandifolia		7	5	6				
Fraxinus pennsylvanica					11			
Juglans nigra		7	11	10	5	9	8	
Liriodendron tulipifera		7	10	14	12	6		
Ostrya virginiana	10	7					6	4
Populus deltoides					13	7		
Prunus serotina	8				8	3	10	
Quercus alba	5	4	6	3				
Quercus bicolor	7	7	2	8			11	7
Quercus macrocarpa				14				
Quercus rubra	3	3	4	4	4	10	5	2
Quercus velutina	6	14			13			
Robinia pseudoacacia		7				11		11
Sassafras albidum					10			
Tilia americana	13	14	9	7			12	6
Ulmus americana			8	9	2	2	4	10
Ulmus rubra			14	10				9
Dead	4	5	3	1	1	1	1	3
