# Supplementary material for: Influence of Derecho and Management Disturbances on Ground-Dwelling Arthropods
Source: Biology (Basel). 2026 Jun 23;15(13):984. doi: 10.3390/biology15130984 (PMC13360023; doi:10.3390/biology15130984)
Supplement: Supplementary file 1 [file biology-15-00984-s001.zip › Wilson_Marshall_Table_S4.pdf]

Table S4. Taxonomic list and count of arthropods collected. Properties have been shorthanded Blue Cast Springs Nature Preserve (BLU), Fogwell Forest Nature Preserve (FOG), Fox Island County Park (FOX), and Hammer Wald Nature Preserve (HAM). This list includes all taxa captured in pitfall traps regardless of occurrence frequency or habitat preference.

			BLU	FOG	FOX	HAM	BLU	FOG	HAM
Class	Order	Family	2024	2024	2024	2024	2016	2016	2016
Arachnida	Acari		9	13	16	4	28	7	8
Arachnida	Araneae		72	21	20	58	87	78	37
Arachnida	Ixodidae				2				
Arachnida	Opiliones		129	60	34	46	38	41	35
Chilopoda			15	4	25	5	2	5	
Decapoda				1					
Diplopoda			1	1	12	3	83	45	61
Insecta	Blattodea	Blattidae					4		
Insecta	Blattodea	Ectobiidae		1		1	7	1	
Insecta	Blattodea						1		
Insecta	Coleoptera	Anthicidae						1	1
Insecta	Coleoptera	Cantharidae		2			3	1	1
Insecta	Coleoptera	Carabidae	30	18	41	55	38	70	26
Insecta	Coleoptera	Cerambycidae		3					
Insecta	Coleoptera	Chrysomelidae		2	21		45	11	33
Insecta	Coleoptera	Cicindellidae	5		10	1	2	1	2
Insecta	Coleoptera	Coccinellidae		1					
Insecta	Coleoptera	Curculionidae		382	583	1	523	532	137
Insecta	Coleoptera	Cydnidae			7				
Insecta	Coleoptera	Elateridae		46	13				
Insecta	Coleoptera	Erotylidae		25		2	526	293	87
Insecta	Coleoptera	Lampyridae		3	2				
Insecta	Coleoptera	Meloidae		1	1				
Insecta	Coleoptera	Mordellidae							1
Insecta	Coleoptera	Phalacridae					18	16	10

Insecta	Coleoptera	Phengodidae				1			
Insecta	Coleoptera	Pselaphidae						1	
Insecta	Coleoptera	Scarabaeidae	4	6	4		15	6	11
Insecta	Coleoptera	Silphidae	3		5	1		2	
Insecta	Coleoptera	Staphylinidae	9	30	30	10	76	77	30
Insecta	Coleoptera	Tenebrionidae	18	18	34	7			
Insecta	Coleoptera								2
Insecta	Collembola	Entomobryidae		4		4	23	15	
Insecta	Collembola	Isotomidae					151	28	8
Insecta	Diptera	Acroceridae			1				
Insecta	Diptera	Anthomyiidae				1			
Insecta	Diptera	Cecidomyiidae						1	
Insecta	Diptera	Culicidae	1	36	7				
Insecta	Diptera	Heleomyzidae					8		1
Insecta	Diptera	Limoniidae		1					
Insecta	Diptera	Lonchaeidae		1		1			
Insecta	Diptera	Muscidae					2	3	2
Insecta	Diptera	Mycetophagidae							3
Insecta	Diptera	Mydidae					1		
Insecta	Diptera	Phoridae					6	2	14
Insecta	Diptera	Rhagionidae					2		1
Insecta	Diptera	Sciaridae						2	
Insecta	Diptera	Simuliidae	2	11	1	2			
Insecta	Diptera	Tephritidae		4	2		22	13	1
Insecta	Diptera	Tipulidae		4	2				
Insecta	Diptera			1				1	6
Insecta	Hemiptera	Cercopidae					1		
Insecta	Hemiptera	Cicadellidae		1			2		
Insecta	Hemiptera	Cicadidae			1	1			
Insecta	Hemiptera	Cydnidae		7	21				
Insecta	Hemiptera	Lygaeidae			1				

Insecta	Hemiptera	Membracidae	1	9	3	2			
Insecta	Hemiptera	Miridae			23				
Insecta	Hemiptera	Mymaridae						2	
Insecta	Hemiptera	Nabidae					3	1	
Insecta	Hemiptera	Pentatomidae		1	4	4			
Insecta	Hemiptera	Reduviidae						3	
Insecta	Hemiptera	Scutelleridae		2		1			
Insecta	Hymenoptera	Apidae			1		1		
Insecta	Hymenoptera	Braconidae					8	4	3
Insecta	Hymenoptera	Eulophidae					2		
Insecta	Hymenoptera	Formicidae	24	47	27	22	457	174	191
Insecta	Hymenoptera	Ichneumonidae		2	2	2			
Insecta	Hymenoptera	Orussidae		6					
Insecta	Hymenoptera	Platygastridae					2		
Insecta	Hymenoptera	Proctotrupidae			1				
Insecta	Hymenoptera	Pteromalidae					1		1
Insecta	Hymenoptera	Scellionidae					1		
Insecta	Hymenoptera	Vespidae					1	3	
Insecta	Hymenoptera								4
Insecta	Lepidoptera	Liparidae		2	2				
Insecta	Lepidoptera	Megathymidae			1				
Insecta	Lepidoptera	Noctuidae		1					
Insecta	Lepidoptera	Tineidae		2					
Insecta	Lepidoptera	Tortricidae			2				
Insecta	Lepidoptera		5	2	1	16			
Insecta	Orthoptera	Acrididae			1				
Insecta	Orthoptera	Gryllacrididae	227	33	3	44	80	33	16
Insecta	Orthoptera	Gryllidae	137	4		21	35	37	31
Insecta	Orthoptera	Hydropsychidae					2		
Insecta	Orthoptera			1					
Insecta	Plecoptera	Pteronarcidae			11				

Malacostraca	Isopoda	448	1176	396	503	517	570	293
