# Supplementary material for: Influence of Derecho and Management Disturbances on Ground-Dwelling Arthropods
Source: Biology (Basel). 2026 Jun 23;15(13):984. doi: 10.3390/biology15130984 (PMC13360023; doi:10.3390/biology15130984)
Supplement: Supplementary file 1 [file biology-15-00984-s001.zip › Wilson_Marshall_Table_S5.pdf]

Table S5. Permutational multivariate analysis of variance (PERMANOVA) of property Bray-Curtis dissimilarity distance matrices post-hoc pairwise comparison results for 2016 and 2024 using *pairwise.adonis2* function in package 'pairwiseAdonis' (version 0.4.1) with a Holm p-value adjustment.

**Year**	**Property Comparison**	**Degrees of Freedom**	**F**	**Adjusted p-Value**
2016	Blue Cast-Fogwell	1,10	2.02	0.073
2016	Blue Cast-Hammer	1,10	5.92	0.012
2016	Fogwell-Hammer	1,10	3.02	0.062
2024	Blue Cast-Fogwell	1,18	8.07	0.006
2024	Blue Cast-Fox Island	1,14	8.59	0.006
2024	Blue Cast-Hammer	1,18	5.84	0.006
2024	Fogwell-Fox Island	1,14	4.84	0.006
2024	Fogwell-Hammer	1,18	4.67	0.011
2024	Fox Island-Hammer	1,14	8.06	0.006
